# Combining default choices and an encounter decision aid to improve tobacco cessation in primary care patients: protocol for a cluster-randomized trial

**DOI:** 10.1186/s12875-022-01859-9

**Published:** 2022-09-24

**Authors:** Christina Hempel-Bruder, Inès Habfast-Robertson, Marie-Anne Durand, Ivan Berlin, Joachim Marti, Yasser Khazaal, Carlos Quinto, Mohamed Faouzi, Kevin Selby

**Affiliations:** 1 Center for Primary Care and Public Health (Unisanté), University of Lausanne, Route de Berne 113, 1010 Lausanne, Switzerland; 2 UMR 1295, CERPOP, University Toulouse III Paul Sabatier, Toulouse, France; 3 Hospital Pitié-Salpêtrière, Paris, France; 4 Department of Addiction Medicine, CHUV, Lausanne, Switzerland; 5 Swiss Medical Association (FMH), Bern, Switzerland

**Keywords:** Provider training, smoking cessation, decision aid, default choice

## Abstract

**Background:**

While quitting smoking dramatically decreases overall mortality, general practitioners (GPs) are less likely to prescribe medications for smoking cessation than other cardiovascular risk factors. Guidelines recommend providers first assess patients’ “readiness” to quit, an “opt-in” strategy, but only a minority of tobacco users are ready to quit on a given day. An “opt-out” strategy offering treatment as the default choice increased quit attempts in hospital and with pregnant women, but has not been tested in primary care. We will assess the efficacy of training GPs to offer treatment as the default choice using an encounter decision aid with current smokers seen in primary care.

**Methods:**

This is a pragmatic cluster-randomized controlled superiority trial with block randomization at the GP level in private practice in French-speaking Switzerland. GPs will be blinded to the arm allocation. The intervention is a half-day training course teaching an ‘opt-out’ approach to smoking cessation using an encounter decision aid (paper or electronic). GPs in the enhanced usual care group receives a brief refresher training about smoking cessation without changing their behaviour. GPs in both arms will recruit 23 patients each prior to routine primary care visits. The primary outcome is the effect of consulting a GP who received the intervention on the 7-day, point prevalence, smoking abstinence 6 months after the baseline appointment. Secondary outcomes include continuous abstinence; number of quit attempts; use of smoking cessation aids; patient-perceived involvement in discussions; and changes in GP behaviour. Patient outcomes will be collected using paper and telephone questionnaires. Assuming 15% drop-out, recruiting 46 GPs with 23 patients each will give us 80% power to detect an increase in smoking cessation from 4% (control) to 10.5% (intervention), with an alpha < 0.05.

**Discussion:**

GP visits are an opportunity to administer proven smoking cessation treatments. We hypothesize GPs offering smoking cessation treatment as the default choice using an encounter decision aid will increase the number of patients who quit. This study could significantly change our approach to smoking cessation in primary care. Default choices and the electronic decision aid are low-cost, easily diffusible interventions.

**Trial registration:**

ClinicalTrials.gov Identifier: NCT04868474, First Posted May 3, 2021, Last Update Posted October 6, 2021.

**Supplementary Information:**

The online version contains supplementary material available at 10.1186/s12875-022-01859-9.

## Background

In Switzerland, over a quarter of the population aged 15 years and older are current smokers. Smoking remains the most important cause of preventable death [1]. Smoking cessation significantly reduces all-cause mortality at all ages [2]. To help with smoking cessation, evidence-based smoking cessation guidelines recommend nicotine replacement therapy (NRT), bupropion and varenicline as first-line therapy in combination with behavioural interventions [3, 4]. General practitioners (GPs) play an important role in smoking cessation promotion by offering advice, treatment and support to their patients. In Switzerland, 60% of smokers expect their doctor to talk about smoking during consultations and 51% desire advice to help them quit smoking [5].

Nonetheless, doctors are less likely to prescribe medication for smoking cessation than for other cardiovascular risk factors [2, 3]. This may be due to a lack of confidence and knowledge in how to prescribe smoking cessation treatments. Doctors lack time and may also consider that discussing smoking cessation is intrusive, that the decision to smoke lies within the private sphere [5–7]. Most smokers attempt to stop smoking without requesting help, partly because of misconceptions regarding the efficacy and potential adverse effects of pharmacotherapies [8].

These barriers are exacerbated by the fact that smokers must “opt-in” to smoking cessation treatment. The current strategy, taught in Switzerland and in most countries, says that quitting advice must be adapted to each patient’s level of motivation to quit smoking using the 5 As model (combining information as well as relational and behavioural techniques). Guidelines recommend that health-care providers first ask patients if they are “ready” to quit, and only offer treatment to those who are ready and desire treatment [9]. This approach limits the reach of tobacco cessation management. Over two-third of Swiss smokers want to quit. However, only a small fraction wish to do so within the next month or two [10]. Hence, most smokers will not be prescribed smoking cessation medications or get behavioural support [9, 11]. Further, clinical trials have shown that smokers who report they are not planning a quit attempt actually quit at similar rates as those who are planning to quit, if they are provided support [9, 12]. Thus, all smokers should be offered treatment using an “opt-out” strategy.

This “opt-in” smoking cessation strategy is in contrast to the treatment of other common chronic medical conditions, such as hypertension and diabetes, where medical treatment is the default option [9, 13]. Accordingly, doctors should offer evidence-based care to all tobacco users without screening for readiness [6]. A before-after study in the UK tested new guidelines advocating “opt-out” quit smoking referrals for pregnant smokers [14]. The default referral approach doubled the number of pregnant smokers setting quit dates and reporting smoking cessation. A before-after study from Switzerland compared intensive cessation visits using motivational interviewing as a default choice for all hospitalized smokers with an acute coronary syndrome [15]. The proactive strategy increased the uptake of smoking cessation counselling and resulted in a non-statistically significant increase in smoking abstinence at 12 months (43% to 51%, *p* = 0.08) [15].

Another means of facilitating the prescription of smoking cessation medications is to use shared decision making (SDM). SDM is an ethical mean of offering quit aids to all current smokers without constraining their autonomy. To achieve SDM, clinicians and patients work together to understand the patient’s condition and determine how best to treat it [16]. SDM is particularly relevant in smoking cessation because medications such as nicotine replacement therapy (NRT), bupropion and varenicline have comparable efficacy but different harms and benefits due to their mechanism of action. A systematic review suggests that DAs may be effective in increasing smoking cessation knowledge, decision quality, and the number of quit attempts [17]. Our research group developed a DA for presenting available pharmacologic interventions for smoking cessation, available in both paper and electronic, online formats. The tool contains information for each treatment option about cost, possible adverse effects. The electronic version also has short animations showing how to use each treatment. The DA was designed to be accessible, intuitive and easy to use. During the consultation, this tool will support GPs in prescribing and help patients chose a pharmacological treatment [18].

The proposed study will therefore test the efficacy of implementing an “opt-out” strategy for smoking cessation treatment using a DA, for current smokers seen in primary care. We will train GPs to offer smoking cessation treatments as the default choice while using shared decision making with an encounter DA (electronic or paper version) to explicitly offer a choice of quit aids. This innovative approach has not been tested in the past and has the potential to increase the number of current smokers seen in primary care who make a quit attempt with a proven quit aid, thereby increasing the number of patients who quit smoking.

### Objectives

The primary objective is to evaluate the effect of a training program encouraging GPs to offer smoking cessation treatment as a default choice to all current smokers using an interactive, encounter decision aid (electronic or paper version), on the proportion of current smokers seen in primary care who have quit smoking 6 months after a baseline visit to their GP, as compared to enhanced usual care.

Secondary objectives are to: 1) evaluate the effect of the intervention on the proportion of patients who make quit attempts; 2) evaluate the effect of the intervention on the proportion of patients who use a quit aid; 3) evaluate the effect of the intervention on patients’ perceived involvement in discussions about smoking cessation; and finally, 4) measure the uptake of key behaviours, notably offering quit aids to all current smokers and use of the DA, by GPs.

## Methods and design

The trial protocol follows the SPIRIT guidelines (Appendix 1) and CONSORT statement for cluster randomized trials.

### Study design and setting

We will perform a cluster-randomized controlled superiority trial with block randomization at the GP level in private practices. GPs will be blinded to the arm allocation. This study is a clinical trial in the category A entailing minimal risks and burdens for participants. The study will be conducted in private practices the French language part of Switzerland.

### Participants and eligibility criteria

We will include a convenience sample of consenting GPs practicing in French-speaking Switzerland. We are focusing on French-speaking Switzerland to facilitate the development of training materials and the decision aid, and to avoid heterogeneity between GPs in French and German-speaking parts of Switzerland regarding their approach to smoking cessation. We aim to be inclusive of GPs at all levels of experience, all practice types, and baseline smoking cessation practices. GPs will recruit current daily smokers seen during routine visits at their practice.

- GP-level inclusion criteria:
  - Private practices located in the Cantons of Vaud, Geneva, Neuchâtel, Jura, Fribourg or Valais
  - Practices with more than 80 individual patients seen in a typical month
  - Willing to recruit approximately twenty smoking patients within a target of six months of the training session
- GP-level exclusion criteria:
  - Having completed a half-day or more of training on smoking cessation within the last 2 years
  - Have plans to retire or relocate outside of French-speaking Switzerland in less than 12 months
- Patient-level inclusion criteria:
  - Consider the GP as their primary care doctor
  - Uses tobacco daily (cigarettes, cigars, smokeless tobacco)
  - At least 18 years of age at the time of inclusion
- Patient-level exclusion criteria:
  - Consulting for an urgent complaint that precludes even a brief discussion of smoking cessation
  - Inability to follow the procedures of the study, e.g. unable to read French-language consent materials, severe psychiatric disorders and dementia.
  - Previous enrolment in a smoking cessation trial within 1 year
  - Current daily use of a pharmacologic smoking cessation aid

### Sample size and power calculation

Based on previous research, we expect approximately 4% of current smokers seen with usual care conditions will report having quit smoking at 6 months after a baseline visit (32). This is based on a model whereby 20% of current smokers discuss a quit attempt (higher than usual with Hawthorne effect) (11), 10% get a specific pharmacologic aid or e-cigarette, and 4% successfully quit. We anticipate a 100% increase in the number of quit discussions in the intervention arm, so about 40% of current smokers discuss a quit attempt. Subsequently, 20% will use a pharmacologic aid or e-cigarette, and 10.5% will successfully quit. For the primary outcome will use mixed effect models accounting for clustered, repeated measures; without adjusting for baseline patient characteristics, we will compare two proportions, using a two-sided test for significance, β = 0.80, α = 0.05, and an intraclass correlation coefficient of 0.03. With these parameters, we need 20 clusters (i.e. individual GPs) in each arm, each with 20 current smokers who consent to inclusion. Assuming 15% drop-out or under-recruitment by individual GPs, we will aim to recruit 46 GPs with 23 patients each, so 1,058 patients. Individual GPs will be capped at 25 patients. No specific interim or safety analyses are planned. STATA version 16.0 for Windows (StataCorp LLC, College Station, USA) will be used for all statistical analyses. Stopping rules for the study: Recruitment of GPs will end when 46 have signed a contract to complete the training program and undergone randomization. Patient recruitment will end when all participating GPs have included ≥ 20 patients and 1,060 patients have been recruited overall. GPs will be considered inactive if they have not recruited patients within the last 6 months. The trial will also end if GP recruitment has ceased and all GPs have completed recruitment. If recruitment is too low (< 200 patient participants 12 months after beginning GP training programs), new recruitment strategies will be put in place and the study may be terminated.

We will conduct semi-structured interviews with a purposive subsample of patients and GPs to better grasp qualitative aspects of their experience with quit aids offered as a default choice and the electronic decision aids. We will aim to recruit a purposive sample of six GPs and ten patients in the intervention and control groups, respectively, or until data saturation. According to Guest et al., a sample of six to twelve participants per group is sufficient to reach thematic data saturation in qualitative interviews [19]. We will thus be guided by this principle but will also check for thematic data saturation before stopping the recruitment of new participants.

### Participant’s timeline

Recruitment of GPs will end when 46 have signed a contract to complete the training program and undergone randomization. Each GP will start the patient recruitment immediately after the training program during approximately the subsequent 6 months. Patient recruitment will end when all participating GPs have included ≥ 20 patients and 1,060 patients have been recruited overall. Patients will be followed during 6 months and GPs during 12 months.

### Randomization

**Fig. 1:**
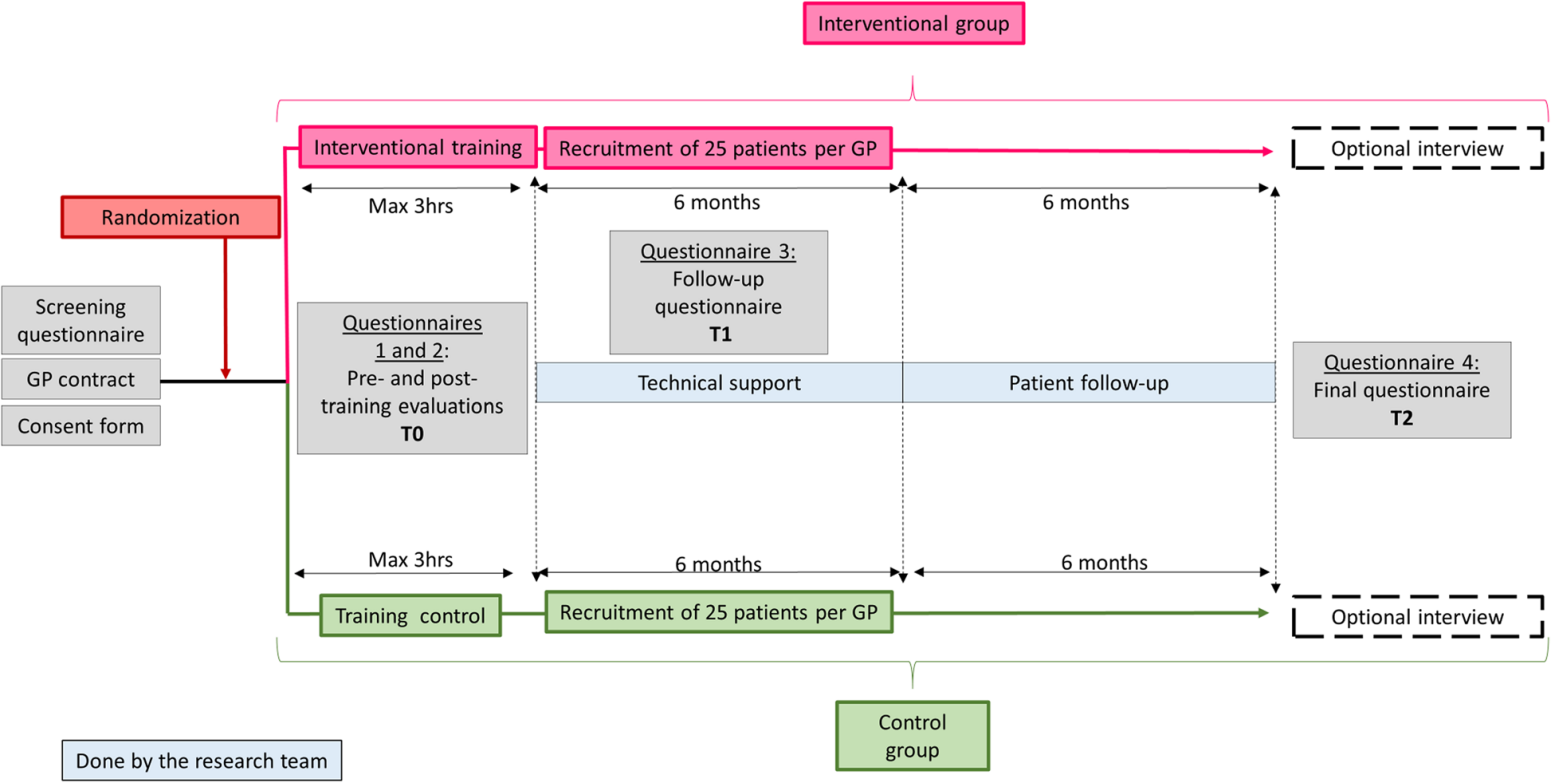
Preliminary CONSORT diagram

The unit of randomization will be GPs who consent to participate in a smoking cessation training program and subsequently recruit eligible patients. The clinical trial protocol follows the CONSORT diagram in Fig. 1. Randomization, done by the study statistician, will be by blocks of two or four GPs to ensure balance between training dates. GPs from the same practice will be randomized together.

### Recruitment

GPs will be recruited by emailing lists from professional organizations, phone calls to private practices, and word of mouth (Appendix 2 shows all documents given to GPs). GPs will then recruit their patients seen for routine visits at their practice (Appendix 3 shows all documents that GPs discuss with their patients).

### Study procedures

**Fig. 2:**
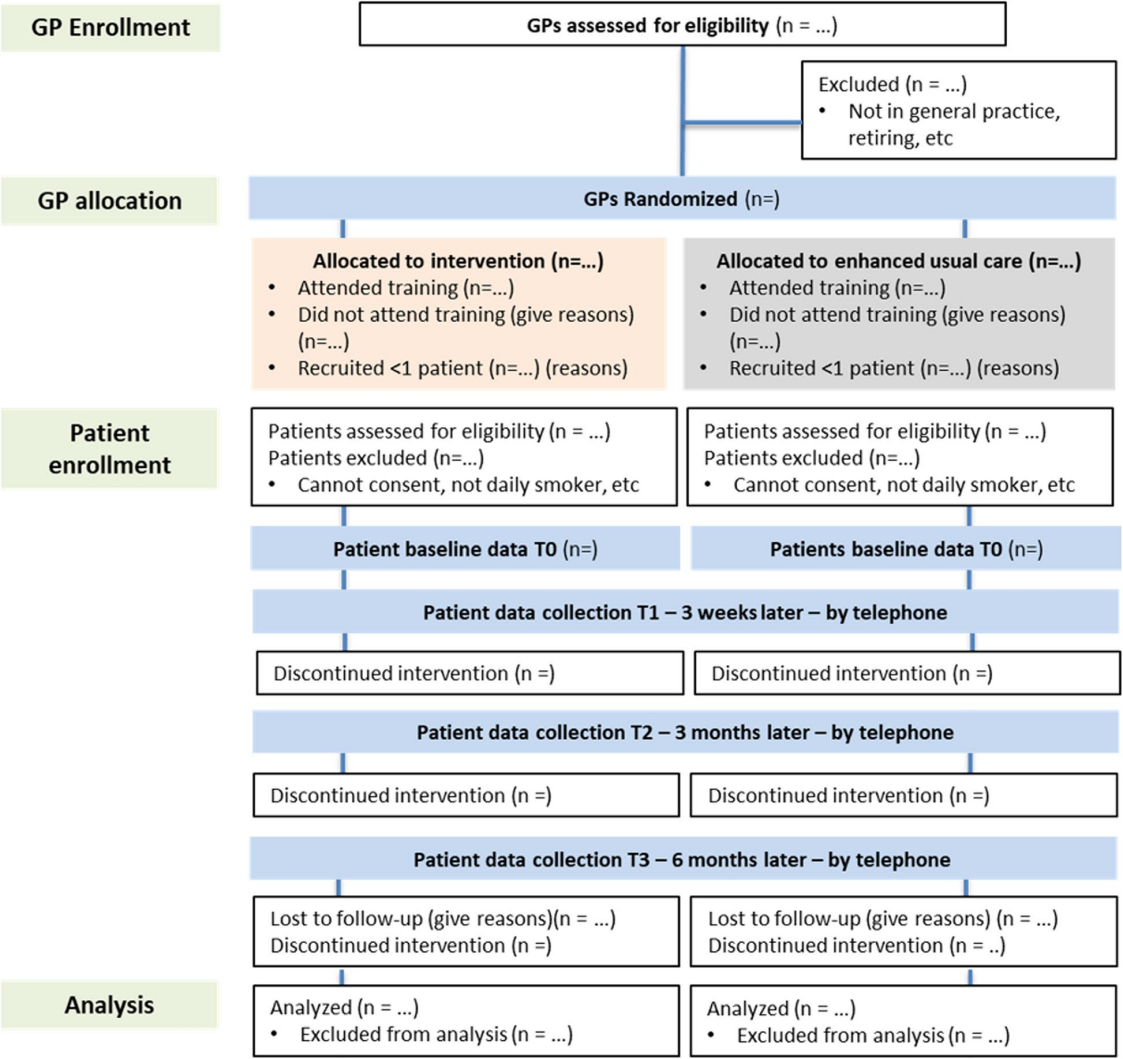
Diagram of expected role of general practitioners

During the recruitment period, the study team will proactively contact GPs to ensure that patient enrolment is going as planned. The GPs will also complete questionnaires at the end of patient recruitment (T1, approximately 6 months after training) and at the end of patient follow-up (T2, approximately 12 months after training). Figure 2 shows the diagram of what is expected by GPs. Moreover, all GPs will also be asked in the T1 questionnaire to participate in a 30-min optional semi-structured interview (see interview guide in Additional file 1; Appendix Table 1). We will perform 10 to 15 interviews, and analyze transcripts using a process evaluation methodology to understand how GPs approach smoking cessation with their patients and how that changed when using default choices and an electronic decision aid.

### Blinding

The smoking cessation experts administering the trainings will not be blinded. GPs will be blinded. Their consent form will only tell them that the study is comparing two versions of a training about smoking cessation, and not the nature of differences between the intervention and enhanced usual care. Nonetheless, GPs in the control group may realize their training is shorter, and GPs in the intervention group will realize their training is significantly different from traditional teachings about smoking cessation. Patients will likewise only be told that the study compares two training programs, and not the precise nature of intervention. Those in the control group should not be conscious that they are receiving usual care, and not quit aids as the default choice or a decision aid. Outcome assessors will not be blinded to study arm when performing follow-up with GPs and patients, however care will be taken not to re-inforce teachings related to the intervention (i.e. use of the decision aid or default choices). The statistician performing the primary outcome analyses will be blinded to group assignments.

### Controlling for contamination and other potential biases

Because this is a pragmatic study, there is a risk of co-administration of interventions and contamination between groups. We will be careful not to reinforce messages about default choices and SDM when contacting GPs about participant inclusion. While cluster-randomized controlled trials limit contamination between groups and better mirror routine clinical practice, they present special challenges not seen in traditional randomized controlled trials [20]. Because the unit of randomization is individual GPs but the unit of analysis is individual patients, there can be imbalances in patient-level confounders. Some clustering is addressed by using the intra-cluster correlation coefficient at the time of analysis. However, bias can come from the post-randomisation recruitment of patients. This bias is addressed by streamlining patient questionnaires, limiting post-randomisation selection bias, and reporting outcomes at both the cluster and individual level [21]. Under-recruitment by certain GPs can exacerbate imbalances, so a study coordinator blinded to treatment assignment will contact participating GPs within two weeks of the training and regularly thereafter to ensure that the inclusion process is functioning at their site.

### Withdrawal and discontinuation

Patient and GP participants can withdraw from the study at any time. Only baseline anonymised information will be retained for analysis once the patient/GP withdraws. If a GP withdraws, his or her patients can still be followed-up individually until the end of the study.

### Intervention arm

The intervention training program will be based on the *Vivre sans tabac* curriculum [6, 7, 20]. The full curriculum has two parts: the first involves a 1.5-h module increasing current knowledge of smoking associated risks and the benefits of pharmacological and non-pharmacological smoking/tobacco cessation aids, modelling the approach to patients at different stages of change (precontemplation, contemplation, and preparation) using video clips. The second part (1-h) involves role-plays practicing the skills learned. In the intervention arm, we will keep the same format (1.5 h interactive module + 1 h of role plays) but will adapt the original content by presenting a quit attempt with a smoking cessation aid as the default to all patients (Table 1) and integrate the use of a decision aid (DA). We will tailor our approach using default choice to various patient scenarios involving current smokers and their reaction to the suggestion of quitting.

**Table 1:** Approach to patient smoker profile types in intervention and enhanced usual care groups

**Smoker profile** (stage in Trans-theoretical model [22])	**Current approach **[5] (control group)	**Approach using default choices and a decision aid** (Intervention group)
25-year-old male smoker seen for flu-like symptoms, smokes ≥ 5 cigarettes per day, more evenings and weekends, uninterested in discussing his tobacco use (pre-contemplative)	“What do you know about the risks of smoking?” “What are potential benefits of quitting?”	“Why don’t you return for an appointment next week so we can follow up your flu symptoms and specifically discuss quitting smoking?” “I understand if you’re not sure about quitting, but I can help. You should give treatment, like with varenicline, a try.”
60-year-old female smoker consulting for a urinary tract infection, 20 cigarettes per day, stressful home and work environment, wants to quit but not now (contemplative)	“What is it that you like and don’t like about cigarettes?” “You say that smoking is bad for your health. What barriers do you see to quitting?” “What do you see as the next step?”	“Now is the time to quit smoking. There effective treatments that can help” “I council my patients to try quitting sooner rather than later” “Let’s make an appointment next week to make a detailed plan”
65-year-old male smoker with mild asthma consulting with seasonal allergies, has already tried quitting and is open to trying again (action)	“Congratulations! I propose setting a quit date.” “Have you hear about medications that can help with quitting? I would recommend that you try one.” “If you’d like, we can schedule regular appointments to help you with your symptoms”	“Congratulations! Let’s set a quit date.” “Nearly all of my patients use a medication to help quit. Some use e-cigs. This tool can help us choose the approach that’s right for you.” “My assistant will call you next week to see if you are tolerating the medication and make a follow-up appointment”

The decision aid will be available in both paper and electronic formats based on our previously developed one-page table (Appendix 4) [18]. It is an encounter DA, designed for use during consultations. The paper version uses a large table, with one row for each pharmacologic aid and one column for each characteristic, such as cost, efficacy and adverse effects. The electronic version (www.howtoquit.ch) expands on the paper version to include larger photos, brief videos demonstrating use of the product, and information on prescribing.

### Enhanced usual care arm

The enhanced usual care arm will consist of a 45-min refresher training about smoking cessation that does not aim to change GP behaviour. It will include the same information about pharmacologic quit aids and electronic cigarettes. Most GPs in French-speaking Switzerland have received this level of instruction about smoking cessation previously, making this training course ‘enhanced’ usual care. We say enhanced because the course will augment knowledge of smoking cessation in the short-term, and participation in the study could trigger more discussions about smoking cessation than routine practice. However, given the proven efficacy of the existing *Vivre sans tabac* curriculum that has been widely implemented in Switzerland [6], it is not ethical to perform a sham training program. Further, it will be impossible to have any blinding between groups without at least some training.

Both groups will be given time to complete questionnaires before and after the intervention. They will be trained to identify current smokers eligible for this trial, complete informed consent, and enrol participants for follow-up.

## Outcomes

**Fig. 3:**
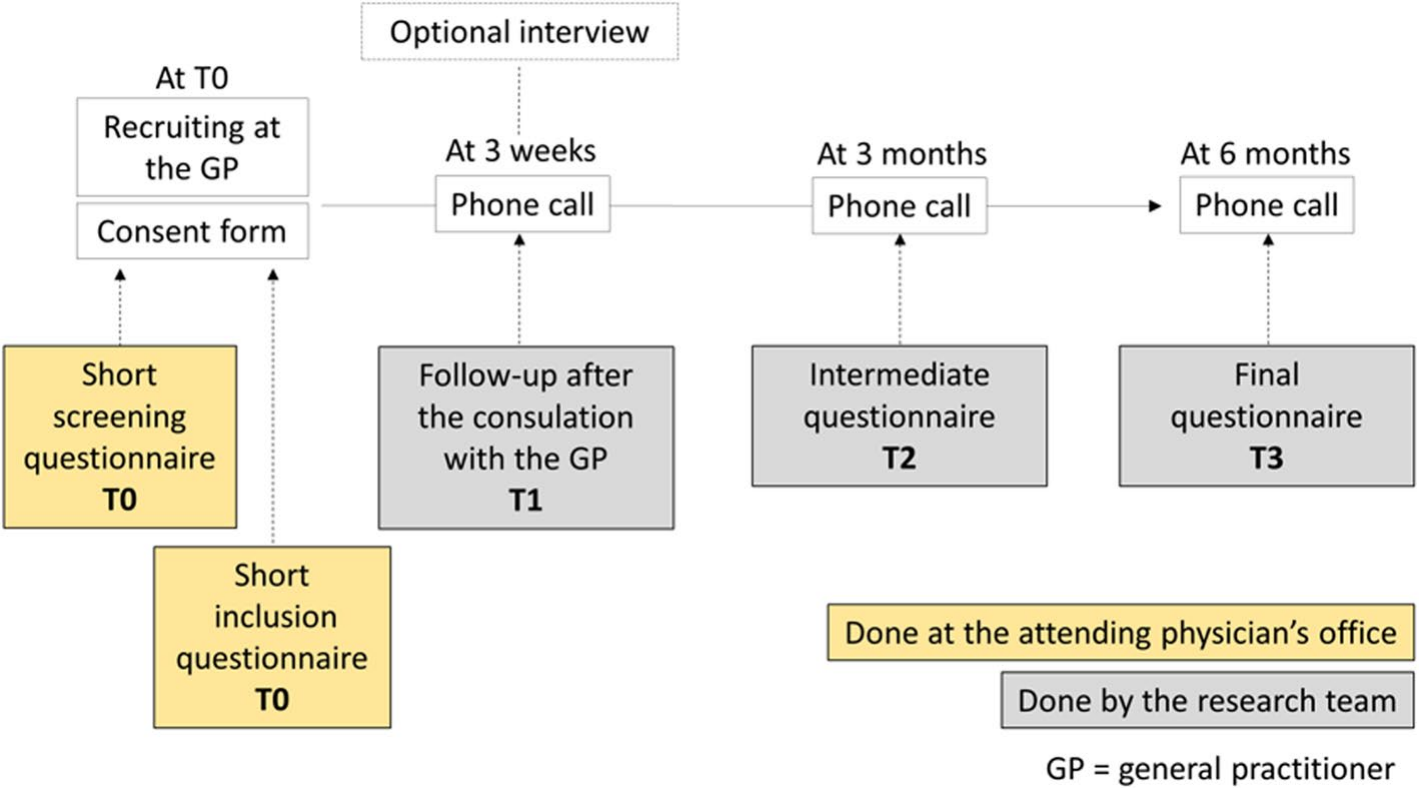
Diagram of the expected role of participating patients

Patients will be followed-up for six months by the research team. A complete list of all patient-level visits, telephone calls and relevant procedures is summarized in Fig. 3.

Follow-up questionnaires at 3 weeks, 3 months and 6 months will primarily be done by telephone, with mailed questionnaires for participants we cannot reach by phone. The content of each questionnaire is detailed in Appendix Table 2.

### Primary outcome measure

The primary outcome measure is smoking cessation. We will measure smoking cessation using the self-reported, 7-day point-prevalence smoking abstinence at six months follow-up after the baseline visit with a GP. Point-prevalence abstinence will be defined as self-reported absence of smoking or use of other tobacco-containing products (smokeless tobacco like iQos, snus, etc.) during the prior 7 days at the time of a follow-up phone call by the research team [21]. The point-prevalence of smoking abstinence at 6 months is a widely used measure in the smoking cessation literature that correlates closely with long-term abstinence. Use of an e-cigarette will not count as tobacco use.

#### Baseline patient and GP factors that may be associated to the primary endpoint

Numerous parameters will be collected from GPs and patients that could influence the difference in smoking cessation between the intervention and control groups. These variables, summarized in Appendix Table 3, will be compared between groups to ensure adequate randomization and allow stratified analyses of the primary result. For patients, we will collect information about socio-demographic characteristics, health literacy, reasons for seeking medical care, smoking behaviours, and baseline motivation to quit smoking. For GPs, we will collect information about socio-demographic characteristics, personal tobacco consumption, practice characteristics, and baseline practices regarding smoking cessation.

### Secondary outcome measures (Appendix Table 4)

- Evaluate the effect of the intervention on the proportion of patients who make at least one quit attempts within six months of their baseline visit
- Evaluate the effect of the intervention on the proportion of patients who use at least one quit aids (pharmacologic, electronic cigarette, or counselling sessions)
- Evaluate the effect of the intervention on patients’ perceived involvement in discussions about smoking cessation
- Measure uptake of key behaviours, notably offering quit aids to all current smokers and use of the electronic decision aid among GPs
- Understand facilitators and barriers to offering patients who smoke smoking cessation aids in primary care, and how default choices and a decision aid influences GP practices

**Fig. 4:**
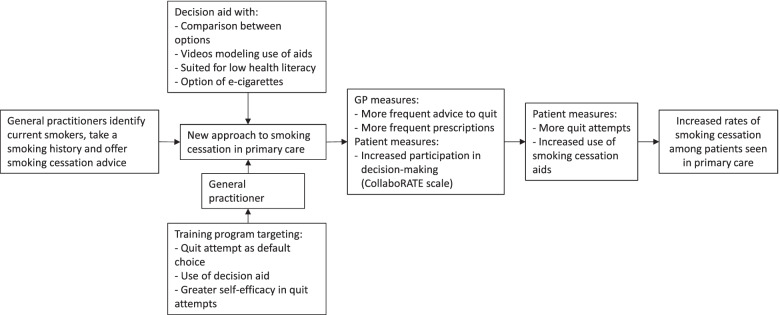
Theoretical framework of interventions to use default choices and shared decision making for smoking cessation in primary care, which will change the approach of GPs to current smokers, increase quit attempts, and eventually increase rates of smoking cessation

Secondary outcome measures will be reported in relation to our theoretical framework (Fig. 4).

## Data collection, management and analysis

GPs and patients will complete several questionnaires throughout the study (see Figs. 1 and 3). To address variable levels of literacy and health literacy of trial participants, we will use validated short form questionnaires whenever possible.

### Data management

We will use REDCap to enter all data collected by GPs (paper forms) and data collected by telephone. REDCap software is a secure software hosted on Unisanté servers that allows for collecting and managing data. Source data will be from paper questionnaires, electronic questionnaires, and audio recordings of qualitative interviews, depending on the data collection point (Figs. 1 and 3). Patient paper questionnaires collected by participating GPs will be sent in batches, bimonthly from GPs to the study team at Unisante, where they will be stored in the Investigator Site File (office of the research assistant) in a closed cupboard. Data will be entered into REDCap Case Report Forms. Data collected during telephone interviews will be directly collected on electronic Case Report Forms in REDCap. Audio recordings from qualitative interviews will be transcribed and then deleted. All documents will be stored for 10 years. All study data will be archived for 10 years after study termination or premature termination of the study. Electronic data will be archived on the Unisanté server. No biological material is being collected.

### Statistical methods

Data will be summarized for all patients and by group: intervention vs enhanced usual care. Continuous and count variables will be summarized as mean (95% confidence interval) or median (IQR) and categorical variables as numbers (percent). Binary outcomes at 6 months will be compared between the two arms intervention vs control using a logistic regression model with clustered-robust standard error estimates. To take into account the clustered repeated measurement design in testing the intervention effect and changes over time, repeated measurement outcomes will be analysed using a mixed-effect models. Depending on the distribution of the outcomes, a logistic regression model will be used for binary outcomes while count regression models (Poisson, negative binomial or zero-inflated negative binomial model) will be used for count outcomes. The intervention x time and others potential interactions will be tested in the models. In addition, subgroup analyses (sex, age, …) will be performed to assess in which subgroup the intervention is most effective. Data analysis will be performed using Stata 16 Software (StataCorp. 2019. Stata Statistical Software: Release 16. College Station, Texas: StataCorp LLC) and performed by a senior Biostatistician.

#### Handling of missing data and drop-outs

For the primary outcome, participants lost to follow-up will be considered as continuing smokers. We do not plan to replace drop-outs as the sample size calculation anticipates a 15% under-recruitment / dropout rate. Imputation will not be done for other variables of interest (weight, laboratory data). Participants lost to follow-up will be considered as continuing smokers without quit attempts/quit aids. For other secondary outcomes, data will be considered missing completely at random. The primary analysis will be an intention to treat analyses, with participants analysed according to their study arm, regardless of the completion rate. Participants lost to follow-up will be considered as continuing smokers.

### Data monitoring and adverse events

This study examines the efficacy of a GP training program and has no medium or high risk factors as defined by the Swiss Clinical Trial Organization (SCTO). Given the low-risk nature of the trial, monitoring will be performed internally at Unisanté by a member of our research support group. We will follow the guidelines for a low-risk monitoring strategy, as outlined by the SCTO. There will be a site initiation visit before inclusion of the first patient and at least one routine monitoring visit after inclusion of the first 2 to 5 patients.

Our intervention is a novel training program for general practitioners. We are measuring the efficacy of this training program and not the efficacy of a specific therapy or medication. All medications prescribed (ex: nicotine replacement therapy) and behavioural interventions administered (ex: advice for quitting and motivational interviewing) are already approved for use and recommended as standard of care in Switzerland. We will not inquire about adverse events or serious adverse events. Further, the information collected from patients is limited to smoking behaviours and does not include sensitive parts of their medical history or diagnoses. The decision aid is not considered a device requiring approval from Swissmedic because it does not make care recommendations or present personalized risk. The decision aid developed presents electronic cigarettes specifically as an option for quitting, as recommended by numerous experts [20] and approved by Swiss law [23].

### Ethics and dissemination

This study is conducted in compliance with the protocol, the current version of the Declaration of Helsinki, the principles of Good Clinical Practice, the Human Research Act (HRA, and the Human Research Ordinance (HRO) as well as other locally relevant legal and regulatory requirements. The Project Leader acknowledges his responsibilities as both the Project Leader and the Sponsor. Our protocol was accepted by the ethics commission of the canton of vaud in February 2021 (Project ID 2020–02,898).

### Protocol amendments

The original ethics approval was received 15.02.2021 for Version 1.2 of all study documents. Since then, we have made amendments to clarify blinding procedures (study staff are not blinded when contacting GPs and patients during follow-up), to clarify the monitoring plan, to allow patients to complete the consent and T0 questionnaire at home and return directly to the central study site, to specify how GPs can withdraw from the study. Approval for V1.4 was obtained 15.02.2022.

### Consent

At the level of GPs: Prior to the training program, GPs will receive an information sheet, complete a consent form and sign a contract outlining reimbursement of time spent in the training program and recruiting patients.

At the level of patients: Patient inclusion will occur at the GP office immediately prior to routine primary care visits. They will receive information materials about the study. If they tell the medical assistant or GP that they wish to participate, they will receive a consent form to sign and after that, will answer a screening questionnaire and a baseline questionnaire.

### Confidentiality

Coded data and participant identification lists will be stored separately. Trial and participant data will be handled with uttermost discretion and made only accessible to authorised personnel who require the data to fulfil their duties within the scope of the study. On the CRFs and other study specific documents, GP and patient participants are only identified by a unique participant number. The participant identification list that links identifying information to GP and patient study codes will be stored on the Unisanté server in a specific folder with access only for the PI and research assistants. The participant identification list will be deleted once patient recruitment and follow-up has been completed. Data from the Unisanté server are regularly backed up. Only allowed study members (doctors, statistician) and scientific collaborators and scientific collaborators with GCP training will have access to data. In case other members join the team (project managers, medical students), they might be given the right to access the data provided they have sufficient training.

### Access to data

Study data will be made publicly available after study completion and initial publication. Requests for study data can also be made by contacting Dr Kevin Selby (kevin.selby@unisante.ch).

### Dissemination policy

The proposed research is highly relevant to primary care physicians, policy makers, the general public and researchers interested in improving tobacco cessation in primary care patients. We hope to maximize implementation potential and determine how this intervention can be sustainably embedded in routine care.

The outputs from this study will be of interest to a wide variety of target audiences, ranging from patient and advocacy groups, healthcare professionals and healthcare organizations, to academics, the Swiss medical society (FMH) and the Federal office of public health, thus maximizing the potential for dissemination. We will work with each target audience to create dissemination and implementation strategies that are tailored to their needs and interests, understandable and pertinent to them. Since the intervention will be freely available, implementation in routine care could be rapid post project completion.

## Discussion

Smoking is the leading cause of preventable death in Switzerland. Adults who quit smoking between 25 and 34 years of age extend their life expectancy by 10 years [24]. Visits to a general practitioner are an opportunity to administer proven smoking cessation treatments, both pharmacologic and behavioural support, that can more than double a person’s chances of quitting successfully. The interventions, such as nicotine replacement therapy and motivational interviewing, have been shown in multiple studies to be safe and cost effective. However, currently few smokers get help with quitting smoking from their physician. We hypothesize that if GPs offer smoking cessation treatment as the default choice to all current smokers using an interactive decision aid, more smokers seen in primary care will quit smoking, as compared to usual care. We will randomly assign GPs to a training program teaching this new approach, which has been tested in the hospital setting and with pregnant women, but never in primary care. If we can improve smoking cessation counselling in primary care, we could have an important beneficial impact on public health.

Our study has several projected strengths, including its pragmatic design, which is very close to routine practice, and an intervention that should be easily implemented if successful. There is an important outcome for patients, which is smoking cessation. The cluster randomized design should also limit contamination between GPs. Our inclusion criteria for GPs and patients are broad so as to maximize the general usability of our results. The GPs who participate should be representative of GPs in private practice in French-speaking Switzerland. We are including poly-morbid and vulnerable patients. We are purposefully keeping our inclusion process brief to encourage less motivated patients to participate and limit co-interventions. A research assistant will follow-up with GPs to ensure they are enrolling patients correctly in the study. During the follow-up calls, we will not reinforce or discuss the concepts presented during the course. We will ensure that all patients are provided with an information sheet and the information that the study is optional.

This study presents several weaknesses, including an incomplete blinding and that the recruitment of patients is realized after the randomization of the GPs. We also use of self-reported smoking cessation at 6 months and not biochemical data to reduce the complexity of the study for the patients. Moreover, we are only intervening with GPs at one time point, and GPs may only see their patients once, such that the intervention may not be sufficiently strong to observe an effect. Another limitation is that the intervention includes two elements (the course and the electronic decision aid) and it will not be possible to know which of the two elements will have induced the most change.

However, we believe that this study has the potential to change significantly our approach to smoking cessation in primary care, and could have worldwide implications. The underuse of smoking cessation aids in primary care has been reported anecdotally and in the scientific literature in multiple countries. Default choices and the electronic decision aid are low-cost, easily diffusible interventions.

## Supplementary Information

**Additional file 1. **

## Data Availability

Study data will be made publicly available after study completion and initial publication. Requests for raw data can also be made to Kevin Selby (kevin.selby@unisante.ch). The decision aids are available free of charge at https://www.unisante.ch/fr/centre-medical/professionnels-sante/aides-decision.

## Abreviations

CO: Carbon Monoxide
CONSORT: Consolidated Standards of Reporting Trials
DA: Decision Aids
EU: European Union
GP: General Practitioner
MA: Medical Assistant
NRT: Nicotine Replacement Therapy
RCT: Randomized Controlled Trial
SDM: Shared Decision Making
UK: United Kingdom
USA: United States of America
