# Supplementary material for: Combining default choices and an encounter decision aid to improve tobacco cessation in primary care patients: protocol for a cluster-randomized trial
Source: BMC Prim Care. 2022 Sep 24;23:246. doi: 10.1186/s12875-022-01859-9 (PMC9508762; doi:10.1186/s12875-022-01859-9)
Supplement: Supplementary file 1 — Additional file 1. [file 12875_2022_1859_MOESM1_ESM.pdf]

17. APPENDIX

Appendix 1: Documents sent to GPs at the time of recruitment (flyer, physician contact information and eligibility questionnaire, information sheet for GPs, consent form for GPs, contract)). Approval obtained for the use of Unisanté and Vivre sans tabac logs.

Appendix 2: Patient documents for recruitment and discussion during consultation with GP (patient contact information, information sheet for patient, eligibility questionnaire, consent form for patient)

Appendix Table 1: Information collected from GPs (interview guide)

Theme	Measure	Variables / questionnaires	Inclusion mailing	Before training	After training	After recruitment (6 months)	One-year follow-up
Time (weeks)				0	0	25	52
Window				NA	NA	±2 months	±2 wk
Format			Paper	Paper	Paper	Electronic	Electronic
Inclusion	Screening	Screening questionnaire	x				
	Study information	Information form	x				
	Signature of informed consent	Consent form	x				
	Contract for reimbursement	Contract from	x				
	Contact	Contact information	x				
Study allocation	Allocation		x				
Demographics	Socio-demographics			x			
	Practice characteristics			x			
	Personal smoking history			x			
Intervention effect	Smoking cessation practices			x	Intended practices	x	x
	Use of default choice				Intended use	x	x
	Use of decision aid				Intended use	x	x
Training program	Training program feedback				x		
Optional interview	Participate in qualitative interview					x	

Appendix 3: The decision aid (paper version). Permission granted for the use of the Unisanté logo.

Appendix Table 2: Summary of data collected from patients

Theme	Measure	Variables / questionnaires	Visit 1 (GP office)	Visit 2 (telephone)	Visit 3 (telephone)	Outcome visit (telephone)	CO verification (research site)
Visit label			V1	V2	V3	V4	
Time (weeks)			0	3	12	24	
Window			NA	±1 wk	±2 wk	±2 wk	
Discontinued	Left study	1 question		x	x	x	
	Reason for discontinuation	1 question		x	x	x	
Inclusion	Screening	Screening questionnaire	x				
	Information	Information form	x				
	Signature of informed consent	Consent form	x				
	Allocation	GP study number	x				
	Contact	Contact information	x				
Demographics	Socio-demographics	Demographic (6 questions)	x				
	Health litteracy	1 question	x				
Medical	Number of chronic medications	1 question	x				
	Reason for consultation	1 question	x				
Smoking	Smoking / vaping history	5 questions	x				
	Intention / motivation to quit	2 questions	x				
	Previous quit history	2 questions	x				
Quit attempt	7-day point abstinence	1 question		x	x	x	x
	Continuous absinence	1 question		x	x	x	x
	Exhaled CO	Test					x
	Made quit attempt?	1 question		x	x	x	
	Use of quit aid	1 question		x	x	x	
	Prescription	2 questions		x			
	Repeat GP quit discussion	1 question		x	x	x	
Consultation	GP discussion (prescription and decision aid?)	4 questions		x			
	Participation in consultation	CollaboRATE (3 questions)		x			
Optional interview	Participate in qualitative interview	1 question		x			
Total number of items (max 40 questions)			18	14	6	6	3

Appendix Table 3: Baseline (at T0) variables collected from General Practitioners (GPs) and patients potentially associated with the primary outcome

**Patient characteristics**	**Question**	**Variable type**
Recruitment date	Date of questionnaire	Continuous
Age	Birth year	Continuous
Gender	Gender	Binary (male / female / other)
Ethnicity	Ethnicity	Categorical
Health Literacy	Comfortable completing medical form	Categorical
Education level	Level of education completed	Categorical
Household revenue	Gross household revenue	Ordinal
Comorbidities	Number of medication classes	Count
Reason for consultation	Reason saw GP	Categorical
Intention to quit smoking	Intention to quit or reduce within the next 3 months	Categorical (stop, reduce, continue)
Motivation to quit smoking	Motivation to quit smoking on a 0 to 10 scale	Ordinal
Tobacco consumption	Number of cigarettes or other tobacco products per day over the last month	Count
Nicotine dependence	Heaviness of smoking index	Ordinal
Previous quit attempts	Number of previous quit attempts	Count
Previous quit methods	Ever use of quit aids	Binary
**GP characteristics**	**Question**	**Variable**
Study training date	Date of questionnaire	Continuous
Study training site	Training site	Categorical
Age	Age in years	Continuous
Gender	Gender	Binary
Years of experience	Year of medical diploma	Continuous
Practice setting	Practice in urban or rural setting	Binary
Size of practice	Number of GPs at same practice at T0	Categorical (<1,2,3, ≥4)
Work time	Work full or part-time	Binary
Country of medical training	Country of medical diploma	Categorical (Switzerland / France / Other EU / Other)
Current smoker (any tobacco product)	Current smoker	Binary
Current vaper	Current vaper	Binary
Former smoker or vaper (any tobacco product)	Former smoker or vaper	Binary
Daily alcohol consumption	Consume alcohol daily	Binary
Marijuana consumption	Consumption in previous 30 days	Binary
Previous training	Training in smoking cessation within the last 5 years	Binary
Baseline practice habits for smoking cessation	Frequency of proposing smoking aids	Ordinal

Appendix Table 4: Summary of secondary outcomes

**Patient-level outcomes**	**Definition**	**Variable type**
Biochemically verified smoking cessation	Exhaled CO ≤ 8 ppm at T3	Binary
Continuous abstinence	Smoking <5 cigarettes total since first quit date at T3	Binary
Smoking cessation at 3 weeks	7-day point prevalence smoking abstinence at T1	Binary
Smoking cessation at 3 months	7-day point prevalence smoking abstinence at T2	Binary
Number of quit attempts	Patient-reported quit attempts at T3	Binary (yes, includes currently abstinent or failed quit attempt /no)
Use of quit aids	Patient-reported use of quit aids at T3	Binary (yes/no)
Participation in smoking cessation discussion with GP	French CollaboRATE scale, adapted to current study	Continuous (0 – 30)
**GP-level outcomes**	**Definition**	**Variable type**
Intention to propose quit aids after training	Intend to propose quit aids to what proportion of patient smokers at T0	Percentage
Proposing quit aids at 6 month follow-up	Propose quit aids to what proportion of patient smokers at T1	Percentage
Maintained proposal of quit aids at 12 months	Propose quit aids to what proportion of patient smokers at T2	Percentage
Intention to use default choices	Intend to use default choices in practice after training program at T0	Binary
Used default choices during the intervention phase	Used default choices and the decision aid at T1	Binary
Maintained use of default choices	Use of default choices after the intervention phase, at T2	Binary
Intention to use the decision aid	Intend to use default choices and the decision aid in practice after training program at T0	Binary
Used the decision aid during intervention phase	Used default choices and the decision aid at T1	Binary
Maintained use of the decision aid	Use of default choices and the decision aid after the intervention phase, at T2	Binary
**Implementation outcomes**	**Definition**	**Variable type**
Recruitment rate of GPs	Number of GPs randomized / number of GPs provided information about the trial	Count
Discontinuation rate of GPs	Number of GPs who complete patient recruitment / number of GPs randomized	Count

Recruitment rate of patients	Number of patients who sign consent / number of patients provided information about the trial	Count
Discontinuation rate of patients	Number of patients who complete 6-month follow-up / number of patients who sign consent	Count

unisanté

Centre universitaire de médecine générale et santé publique · Lausanne

Vivre sans TABAC

Training dates

CHUV :THURSDAY, 9am to 12pm

20.01.22

03.02.22

17.02.22

We need your help!

FIRST STUDY

Measuring the effectiveness of a new approach to "vIvRe Sans Tabac" training for treating physicians on the rate of smoking cessation of their patients

DESCRIPTION

- Training for general practitioners to **improve the management of smoking patients** and increase their quit rate
- Applies **to all smoking patients**, regardless of their level of motivation

For more information **(see overleaf) please** contact us at etude.first@unisante.ch

STAKEHOLDERS

- Dr Kevin Selby (079 556.67.53)
- Dr. Christina Hempel-Bruder
- Dr. Ines Habfast-Robertson
- Dr. Anne Boesch

In partnership with the Federation of Swiss Physicians (FMH)

unisanté

Centre universitaire de médecine générale
et santé publique · Lausanne

Training dates

<u>CHUV</u> :THURSDAY, 9am to 12pm

20.01.22

03.02.22

17.02.22

<u>YOUR INVOLVEMENT</u>

- Participate in **3 hours of training** (for the smooth running of the randomisation, please indicate 2 possible dates)
- After the training, over a period of 6 months, **recruit 20 patients** from your patient base
- The recruited **patients** will be **followed by telephone by our** research **team**
- At the end, you will receive **feedback** on the proportion of your patients who have managed to quit smoking

<u>INDEMNIFICATION</u>

Your time is compensated with **150 CHF/hour**
(6 to 8 hours in total, maximum 1200 CHF)

Other training dates will follow! Please check our website or let us know if you are interested by email/phone. We will keep you informed of the dates.

<u>REGISTRATION</u>

You can register by e-mail **etude.first@unisante.ch** or directly on the study website: **https://www.unisante.ch/etude-first**

Study ID (to be assigned by the research team) :____

1. CONTACT DETAILS OF THE ATTENDING PHYSICIAN

Please provide your contact details below:

Last name, First name: ....

Address of the medical practice:

.... Telephone number of the

medical practice: ....

E-mail address:

.... In case of

questions or reminders, you prefer to be contacted by : ☐ ☐

☐ E-mail, Telephone, Mail

2. ELIGIBILITY QUESTIONNAIRELocation ____________________, date : ____________________

Dear colleague,

Here is a short questionnaire to see if you are eligible to participate in a tobacco study. If you are eligible and interested in participating in a study, please copy the consent form and the contract (regarding financial compensation).

Participation in this study is voluntary and no explanation is necessary if you refuse.

Please answer the following questions:

Inclusion criteria for treating physicians	YES	NO
Do you work in a practice located in the canton of Vaud, Geneva, Fribourg, Jura, Neuchâtel or Valais?		
In a standard month of consultation at your practice, did you see > 80 patients?		
**If the answer is NO to ≥ 1 of the 2 questions above STOP**		
Exclusion criteria for referring physicians	YES	NO
In the last two years, have you participated in a comprehensive *Tobacco Free* training course (> 2 hours of training)?		
In the next 12 months, do you intend to retire or move to a non-French speaking area of Switzerland?		
**If the answer is YES in ≥ 1 of the 2 questions above STOP**		

If you have any questions or comments, we will be happy to provide you with further information.

Contact: etude.first@unisante.ch

Dr Kevin Selby, +41 79 556 6753

INFORMATION SHEET FOR TREATING PHYSICIANS

Study on the proportion of patients quitting smoking after 6 months of follow-up with their GP, even though the GP has participated in the *Living Without Tobacco* training	
Principal Investigator	Dr med, Kevin SELBY (Unisanté, Route de Berne 113, 1010 Lausanne, +4179 556 67 53)
Ethics Committee number	2020-02898

Dear colleague,
We would like to invite you to participate in our study in the field of smoking cessation. This information sheet describes the details of our study and the role you could play in it.

Aim of the study :
We are interested in the proportion of patients who quit smoking within 6 months of an appointment with their GP, after the GP has participated in the *Living Without Tobacco* (LWT) training. This currently exists in two forms and an update is currently under development.

VST is a continuing education course that offers practising physicians a qualification in smoking cessation based on medical knowledge and communication skills. The course has been in existence since 2002 and has already provided thousands of practising physicians with continuing medical education courses on smoking cessation counselling and prescribing. A randomised controlled trial has also shown that this training improves the smoking cessation skills of doctors in training (physician assistants), as well as the number of patients quitting smoking at 1 year. For our study, we are working closely with the Swiss Medical Association (FMH), which is the organisation responsible for the VST programme.

Our study is financed by the Tobacco Prevention Funds. Study

design :
We are conducting a randomised controlled trial with treating physicians (TMs) from the French-speaking region of Switzerland, as well as their patients. The GPs will be randomised in a single blinded fashion into the control group (who will receive the control training) or the intervention group (who will receive the intervention training).

Role of the recruited TMs:
The recruited MVs will first participate in the VST training which will take place over half a day (maximum 1-3 hours). On the same day, they will also be briefed on the study process and patient recruitment.

Upon completion of the training and for the next 6 months, the TMs will be asked to recruit 25 (maximum) patients each (all adults, daily smokers, and who are seen at their consultation for non-urgent reasons). The patients will then be followed up for 6 months by the research team. TMs will be asked to complete a total of 4 questionnaires during the study (one before VST training, one after VST training, one at the end of recruitment and one after 12 months (see Figure 1). In addition, an optional interview (5th questionnaire) will be offered at the end of the study, which the TMs can refuse to answer.

General information about the study :

TMs who agree to participate in the study will be randomised (single-blind) to either the control or intervention group. As mentioned above, the TMs will then participate in the VST training and receive information about the study process and patient recruitment. They will then be asked to complete four questionnaires with questions on socio-demographics, personal smoking habits, education and smoking cessation.

Patient recruitment will start directly after the VST training and will continue until each TM has recruited 25 patients (minimum 20) from his or her patient base (we estimate that this will take place over a period of 6 months; see figure 1). Each participating TM will receive a complete file containing the documents to be sent to the patients (see table 1).

Recruitment will take place directly at the TM's office, during non-urgent consultations (see Figure 2). It can be done directly by the GP during the consultation, or started by the medical assistant before the consultation. The aim is to give patients an information sheet. This information sheet should enable participants to make an informed decision about participating in the study. It should be noted that patients will be able to ask questions either to the caregivers on site or by telephone to a trained research assistant. Each participant will be informed that their participation in the study is voluntary and that they can refuse to participate or withdraw from the study at any time, and that this will not affect their medical follow-up. Participants will then have up to 15 minutes each to decide whether or not they wish to participate in the study. Those who wish to participate will sign the consent form. Note that the consent form will also need to be signed by the GP on behalf of the study investigators and a copy of the signed informed consent will be given to the study participant.

Patients who ask for more time to think can take the relevant papers (information sheet, consent, screening questionnaire, inclusion questionnaire) home. If this is the case, the patient has 14 days to make a decision, after which he/she can either take the completed papers back to his/her GP who will co-sign the consent at that time, or the patient sends all the papers back to the research team directly with the pre-stamped envelopes provided.

Patients who have signed the consent form will then be asked to complete a screening questionnaire to see if they are eligible for the study. Those who meet the inclusion criteria but do not have any exclusion criteria will be included in the study and will be able to complete the first questionnaire of the study on their drinking habits. Note that both these questionnaires (screening and inclusion) will have to be completed on the day of the consultation where recruitment will have taken place (if not at home as described above).

The doctor can then put all the papers (consent, screening questionnaire, inclusion questionnaire) in the stamped envelope that will be provided on the day of the training. Ideally, you can send us one envelope per week (address: FIRST Team, Dr Kevin Selby, Route de Berne 113, 1010 Lausanne). Please note that one envelope can contain several patient files if they are separated by the plastic covers with which they were provided. Note that the various sheets/forms mentioned above that relate to patients are stapled together (except for the information sheet which the patient must take with him/her) and that the staple should NOT be removed for photocopying, etc.

The patients included will then be followed for a period of 6 months by the research team (see figure 3). The team will contact them by telephone (or by e-mail in the case of unreachable patients) at

three times (at 3 weeks, 3 months and 6 months). The telephone interviews will last approximately a maximum of 15 minutes each and the questions asked will be about their smoking habits and their attempts and motivation to quit.

It should be noted that a study coordinator will be in contact with the TMs during the two weeks following the VST training to ensure that recruitment is progressing and to answer any questions the TMs may have.

Figure 1: schéma du rôle des MT recrutés

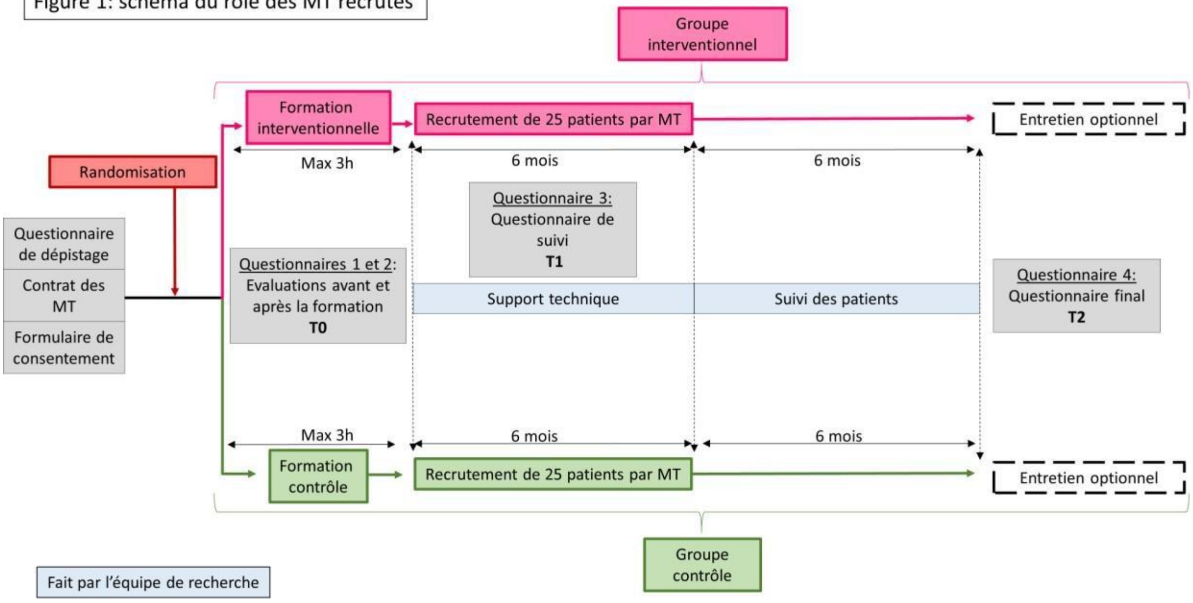

Figure 2: Protocole d'inclusion des patients

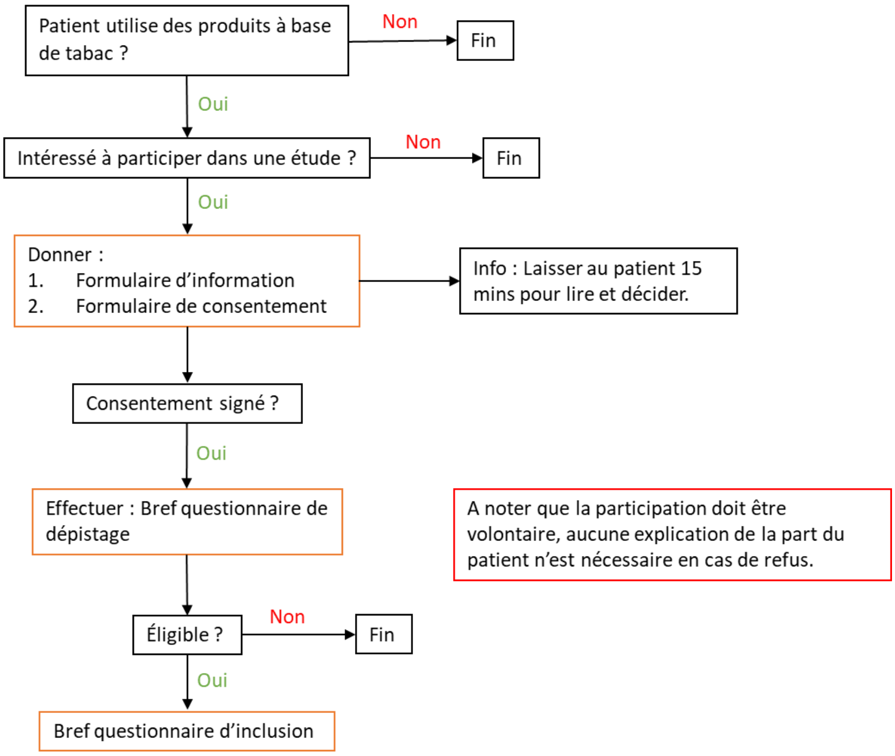

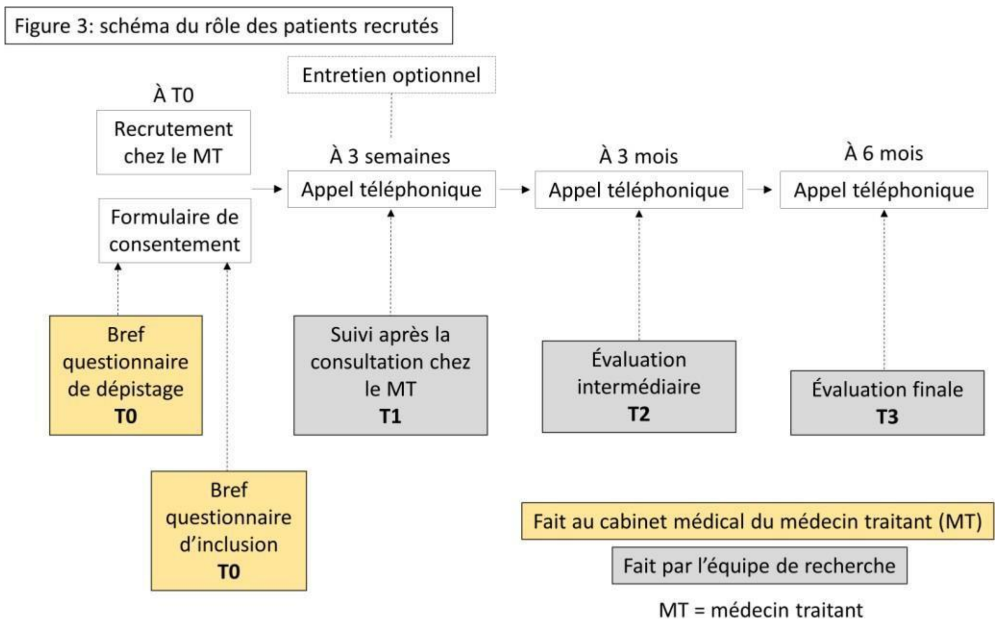

Table 1: Complete records to be given to patients on the day of recruitment (4 documents)

**Information sheet**	It should enable participants to make an informed decision about whether to participate in the study
**Consent form**	Participants will have 15 minutes each to decide whether or not they wish to participate in the study. Those who wish to participate will then sign the consent form. This should also be signed by the MT on behalf of the study investigators A copy of the signed informed consent will be given to the study participant<br><br>N.B.: The signed form will be returned to us in a stamped envelope (approx. 1x/week)
**Screening questionnaire**	Questionnaire to see if patients are eligible for the study or not.<br>This must be completed on the day of the consultation where the recruitment is made
**Inclusion questionnaire**	1st questionnaire for patients who will meet the inclusion criteria without presenting exclusion criteria<br>This must be completed on the day of the consultation where the recruitment is made

Benefits for study participants :

For the TMs, it is an opportunity to participate in the VST training free of charge and potentially improve their knowledge and skills in the field of smoking cessation. At the end of the study, the TMs will also receive a report informing them of the proportion of their patients who have made a quit attempt during the 6 months of follow-up and the proportion who have successfully quit.

For patients, there are no direct benefits. However, an improvement in overall knowledge in the field of smoking cessation could improve the quit rate in future patients.

Financial compensation for participants:

The recruited TMs will be reimbursed up to CHF 1'250 each, as compensation for the time spent on VST training and patient recruitment. More detailed information is available in the attached document (see Appendix, Contract for treating physicians). For those who will also participate in an optional interview, there is an additional compensation of 50 CHF for approximately 15 minutes of your time.

Patients will not receive any compensation (financial or otherwise) except for those who participate in an optional interview; these will receive a financial compensation of CHF 50.

Risks:

There are no risks associated with participating in the study

Participants' rights:

TMs are free to accept or refuse to participate in the study, as will be their patients. They do not need to justify their decision if they refuse to participate. TMs can also ask the research team (etude.first@unisante.ch, +4179 556 67 53) any questions they may have about the study, even after the study has started.

Obligations of the participants :

The TMs who agree to participate in the study must undergo the full VST training (the maximum duration is 3 hours) as well as the explanation of the study and the recruitment of patients. Each TM will then have to recruit 25 patients (maximum) according to the inclusion criteria. In addition, they will also have to answer 4 questionnaires (2 during the VST training (one before and one after), one at 6 months and one after 12 months). The questions will include personal smoking habits, education and socio-demographics.

The TMs will receive a contract detailing their commitment to be randomly assigned (to the control or intervention group), to recruit patients and to complete questionnaires.

Withdrawal of the project :

TMs may withdraw from the study at any time during the study. To do so, they must inform a member of the research team. Please note that they will be asked for the reason for withdrawal. By withdrawing from the study, the participants agree that the data collected so far will be analysed anonymously.

Data privacy :

The data (the answers to the questionnaires) will be collected in a completely anonymous way. We have no way of matching the responses with the identities of the TMs involved. Indeed, we will not collect any personal data that could allow direct identification of the TMs. The data will be stored and archived under the responsibility of Dr Kevin Selby without time limit.

Contact details of the research team :

If you need to contact Dr Kevin Selby, the research leader, here are his contact details: telephone number (+41795566753) or email address (kevin.selby@unisante.ch). Alternatively, you can reach the research team at the following email address, etude.first@unisante.ch.

CONSENT FORM FOR TREATING PHYSICIANS PARTICIPATING IN THE "FIRST" RANDOMISED STUDY

Declaration of consent

Written consent statement for participation in a research project

Please read this form carefully. Do not hesitate to ask questions if you do not understand something or if you need clarification. Your written consent is required to participate in the project.

**BASEC number of the research project (after submission to the relevant ethics committee) :**	2020-02898
**Title**<br>**(scientific and usual) :**	FIRST: The effectiveness of the "vIvRE Sans Tabac" training of treating physicians on the rate of cessation of smoking by their patients
**Responsible institution**<br>**(project leader and full address) :**	Unisanté<br>University Centre for General Practice and Public Health<br>Route de Berne 113<br>1010 Lausanne
**Location :**	Lausanne
**Responsible for the project on the site :**<br>Printed name and surname :	Dr Kevin Selby
**Participant :**<br>Printed name and surname: Date of birth :	

- I declare that I have been informed orally and in writing by the undersigned physician-investigator of the objectives and course of the research project as well as the possible advantages and disadvantages and the possible risks.
- I am taking part in this project voluntarily and I accept the contents of the information sheet I have been given on the above-mentioned project. I have had sufficient time to make my decision.
- I have received answers to the questions I asked in relation to participation in this project. I keep the information sheet and receive a copy of my written consent.
- I agree that the competent specialists of the project management and the competent ethics commission may consult my uncoded data in order to carry out

This is subject to the strict confidentiality of such data.

- I can revoke my consent to participate in the project at any time and without having to justify myself. The data collected up to the withdrawal will however be analysed in the framework of the project.

Place, date	Signature of the participating physician

Certification by the investigating physician: I hereby certify that I have explained to the participant the nature, importance and scope of the project. I hereby declare that I have fulfilled all my obligations in connection with this project in accordance with applicable Swiss law. Should I become aware of any problems at any time during the implementation of the project,
I will inform the participant immediately if I become aware of any factors that may affect his/her consent to take part in the project.

Place, date	Name and surname of the investigating doctor<br><br><br>Signature of the investigating physician

CONTRACT FOR TREATING PHYSICIANS

<u>Contract for the participation of treating physicians in the FIRST study</u>

We want to reward you for the time you spend on this study. You will be

reimbursed according to the following scale:

Description	Max per unit	Max number	Reimbursement total
Participation in the *Living Without tobacco* and questionnaire responses	200 CHF	1	200.00 CHF
Recruiting a patient and filling in the application form<br>consent	30 CHF	25	750.00 CHF
Full recruitment of the 23 patients (max 25) and responses to the 6-month questionnaire	300 CHF	1	300.00 CHF
		Total	CHF 1,250.00

The consent forms received by the research team will refer to the number of patients included.

An electronic questionnaire will be sent to you at the end of the recruitment of the 23 patients and at least 6 months after your training.

You will also be reimbursed for travel costs for the *Living Without Tobacco* training (mileage or public transport) and parking.

Reimbursement will be made by bank transfer every 3 months and at the end of the questionnaire at 6 months follow-up.

Please provide your bank details below.

IBAN: ....

Account name: ....

Bank address: ....

Physician participating in the study :

Last name, First name: ....

Signature:
.... Date:
....

Representative of the research team :

First name, Last name: ....

Signature: ....
Date: ....

T0 Screening questionnaire

Record ID : __________________________________

The date on which the questionnaire was completed : __________________________________

Madam, Sir,

You have recently been informed about our smoking cessation study. You have agreed to participate and we thank you for that.

Here is a short questionnaire to find out if you can participate in our study, in which your doctor is already involved.

1. Is the doctor you are seeing today<br>Is your GP (or family doctor)?	◯ No<br>◯ Yes
2. Do you smoke every day?<br>conventional cigarettes, cigars, cigarillos<br>smokeless tobacco products*)	◯ No<br>◯ or
3. Are you 18 years or older?	◯ No<br>◯ Yes
4. Can you read and respond to<br>written questions in French?	◯ No<br>◯ Yes

If the answer is NO to 1 of the 4 questions above, you can stop the questionnaire and return the sheet to your doctor and/or medical assistant.

5. Are you consulting for an emergency that,<br>in your opinion, makes it impossible to talk aboutYes<br><br>en briefly) about tobacco?	◯ No<br>◯<br>(ev
6. Have you ever taken part in a study on<br>on the subject of smoking in the last year?	◯ No<br>◯ Yes
7. Do you regularly take treatment for<br>help you stop smoking (e.g. a<br><br>otine substitute<br>Champix or Zyban)?<br><br>(electronic cigarettes do not count)	◯ No<br>◯<br>nic<br>,

If the answer is YES in 1 of the 3 questions above you can stop the questionnaire and return the sheet to your doctor and/or medical assistant.

Lexicon :

- Smokeless tobacco products* => chewing tobacco, snus, Iqos

T0 Inclusion questionnaire

Record ID : ____________________

The date on which the questionnaire was completed : ____________________

1. You are:
   - ◯ A man
   - ◯ A woman
   - ◯ Other

2. What year were you born? ____________________

3. In which country were you born?
   - ◯
   - ◯ S
   witze
   rland
   Other

4. How many years have you lived in Switzerland? ____________________

5. What nationality are you?

   (Multiple answers possible)
   - ☐ Switzerlan
   - ☐ d Other

5.a. If other; what nationality(ies) are you? ____________________

6. Are you comfortable filling in a questionnaire yourself? medical form?

   (e.g., a questionnaire about your health and your history when you go to a new home doctor)

   (Only one answer possible)
   - ◯ Always
   - ◯ Often
   - ◯ Sometimes
   - ◯ Rarely
   - ◯ Never
   - ◯ I don't know

7. What is your highest level of education? Compulsory

   (Only one answer possible)
   - ◯ school or less
   - ◯ Apprenticeship
   - ◯ High school diploma
   - ◯ High school or university
   - ◯ I don't know

8.
household's
order of magnitude, less than$20,000gross*?

(Only one answer possible)

What isyour
- ◯ annual income, in
- ◯ From 20,000 to 50,000
- ◯ From 50'000 to 80'000
- ◯ From 80'000 to 120'000
- ◯ francs 120'000 francs
- ◯ and more
- ◯ I do not wish to answer I do
not know

9. What medicines do you take on a daily basis?<br><br>(Multiple answers possible)	☐ Anti-platelet/anti-coagulant (e.g. aspirin, sintrom)<br>☐ Antihypertensive<br>☐ Anticholesterol<br>☐ Antidiabetic<br>☐ Hormonal treatment (e.g. thyroid, contraceptive, estrogen)<br>☐ Painkillers<br>☐ Inhaler* (for asthma or bronchitis)<br>☐ Antidepressant<br>☐ Sleeping pill or relaxant<br>☐ Other<br>☐ I am not taking any treatment
9.a. If other, what medication are you taking?	______________________________
10. What tobacco product do you use on a daily basis?<br><br>(Several answers possible)	☐<br>☐ Conventional cigarettes Cigars/cigarios<br>☐ Pipe<br>☐ Heated Tobacco*<br>☐ Snus<br>☐ Shisha<br>☐ Other
10.a. If other; what tobacco product(s) do you use?	______________________________
11. a. In general, when I wake up in the morning, within 5 minutes<br><br>(Only one answer possible)	◯<br>◯ I smoke my first<br>◯ cigarette:Within 6-30<br>◯ minutes Within 31-60<br>minutes<br>After more than 60 minutes
11. b. On average, I smoke per day:≤<br><br>(Only one answer possible)	◯ 10 cigarettes<br>◯ 11-20<br>◯ 21-30<br>◯ ≥ 31
12. Do you intend to quit smoking in the next few months?<br><br>(Only one answer possible)	◯ No<br>◯ Next 3 months? Yes, I intend to try to<br>◯ stop No,but I intend to reduce my<br>consumption◯<br>I don't know
12. a. If yes, on a scale of 0-10, how motivated are you to quit? (0 = no motivation; 10= very motivated)<br><br>(Only one answer possible)<br><br>◯ 0 ◯ 1 ◯ 2 ◯ 3 ◯ 4 ◯ 5 ◯ 6 ◯ 7 ◯ 8 ◯ 9 ◯ 10	
13. In the past year, have you ever done any attempts to quit smoking?	◯ No<br>◯ Yes
13.a. If yes, how many attempts to stop did you do?<br><br>(Only one answer possible)	◯ 1<br>◯ 2<br>◯ 3<br>◯ 4<br>◯ > 4

14. Have you ever tried any treatment or medication?<br>method to help you stop smoking?<br><br>(Example: nicotine substitute, varenicline,<br>bupropion, electronic cigarette, other)	◯ No<br>◯ Yes<br>◯ I don't know
14.a. If yes, please specify:	______________________
15. In the past month, have you used a<br>nicotine containing product (WITHOUT tobacco)?<br><br>(Of type: vapour/electronic cigarette, heated<br>tobacco products, other)	◯ No<br>◯ Yes
15.a. If yes, please specify which one(s):	______________________
16.<br>to your GP fora check-up	◯ What is the reason for your visit<br>◯ today?A follow-up<br>◯ An emergency potentially related to tobacco<br>◯ (Only one answer possible)A<br>◯ prescription renewalOther
16.a. If other, please specify the reason:	______________________

Lexicon :

- The gross annual income* => this is the sum of all income received by the household from activities (wages, salaries, unemployment, overtime, pensions received, rental income from real estate, commercial and non-commercial profits, agricultural profits). No expenses have yet been charged.
- Inhaler* => a medicine that is taken by mouth when inhaled
- Heated tobacco* => example: Iqos
- Snus* => sucking tobacco, sachet that is put in the mouth.

PATIENT INFORMATION SHEET

Study on the proportion of patients quitting smoking after 6 months of follow-up with their doctor, even though the latter had participated in the *Living Without Tobacco* training	
Principal Investigator	Dr med, Kevin SELBY (Unisanté, Rue de Bugnon 44, 1011 Lausanne, +41795566753)
Number of the Ethics Committee	2020-02898

Dear patient,
You have been identified as eligible to participate in our study in the area of smoking cessation. We would like to invite you to participate. This information sheet describes the details of our study and the role you could play in it. This information should enable you to make a free and informed decision about whether or not to participate in the study.

<u>Aim of the study :</u>
GPs play an important role in smoking cessation. In this study, we test two versions of a smoking cessation training course offered to established GPs. We are interested in the proportion of patients who quit smoking within 6 months of a consultation with their GP, after their GP has attended one of these courses. Your GP has recently attended one of these two courses and is participating in our study.

<u>Study design :</u>
This is a randomised study, i.e. with a random allocation of these two training courses to the treating physicians, allowing to avoid several biases, in particular selection biases. Our study focuses on treating physicians in the French-speaking region of Switzerland and their patients.

<u>Role of the patients recruited :</u>
If you agree to take part in the study, you will receive a consent form which you will have to sign and return to your GP on the day the study is presented to you. If you need more time to think about it, you can take the important papers (information sheet, consent, eligibility questionnaire, inclusion question) home. Afterwards, you have two options, either to return to your GP with the study papers who will countersign them and send them to the research team. Or you can send all the papers (in the pre-stamped envelope given to you) directly to the research team. If you send the papers directly to the research team we will call you back to make sure you have no further questions about the study.

Please note that participation in the study is voluntary. You have the possibility to ask questions to your doctor or to the research team by phone (+41795566753). If you refuse to participate, you do not need to provide any explanation and it goes without saying that there will be no consequences for your medical care.

If you agree to take part in the study, you will first complete an initial screening questionnaire (which will confirm that you are eligible for the study) and then a short inclusion questionnaire about your smoking.

Then, you will be followed up by the research team for 6 months (*Figure 3*), through three telephone contacts (or by email in case of no answer) during which you will be asked questions. The three telephone interviews will take place at T1 (3 weeks), T2 (3 months) and T3 (6 months).

months) and will each last approximately 10 minutes. The questions asked will include your smoking habits and your attempts and motivation to quit.

Please note that you will also have the possibility to participate in an optional interview (additional interview, about 20 minutes), which will take place at the same time as the first phone call (at 3 weeks).

Figure 3: schéma du rôle des patients recrutés

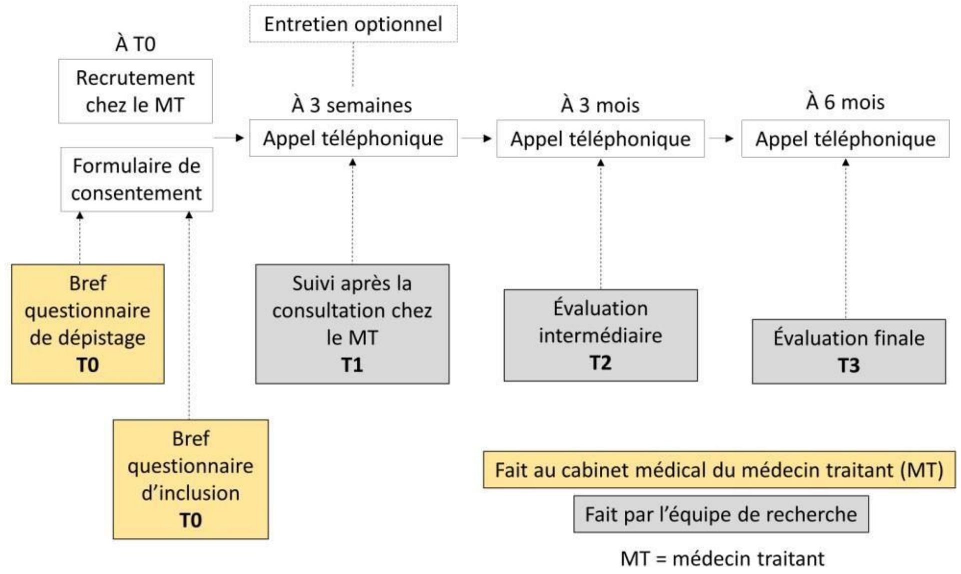

Benefits for study participants :

There are no direct benefits. However, an improvement in the overall knowledge in the field of smoking cessation could improve smoking cessation rates in future patients.

Financial compensation for participants:

You will not receive any compensation (financial or otherwise) unless you participate in the optional interview (compensation of 50.-).

Risks:

There are no risks associated with participating in the study

Participants' rights:

You are free to accept or refuse to participate in the study. You do not have to justify your decision and you can ask questions at any time.

Obligations of the participants :

If you agree to participate in the study, you agree to be followed up for 6 months by the research team through 3 telephone contacts (at T1 (3 weeks), at T2 (3 months) and at T3 (6 months)). All the information collected will be done by telephone (or by e-mail if you cannot be reached by telephone). The questions you will be asked to answer are about your smoking habits and attempts to quit.

Data privacy :

Identified information, including names, addresses and telephone numbers, will be stored

by the research team to enable them to contact you on three occasions. All this information is confidential and will only be accessible by the research team concerned.

The data (the answers to the questionnaires) will be collected in a completely anonymous way. We have no way of directly linking the answers to your identity. Your GP will know how many of his patients will have quit smoking within 6 months of the consultation, but will not have access to your answers (to the questionnaires). The data will be stored and archived under the responsibility of (Dr Kevin Selby, +41795566753) without time limit.

Withdrawal from the study :
You can withdraw from the study at any time. To do so, you can inform your doctor, the research team during follow-up phone calls (or email) or call the research team (+41795566753).

If you wish to withdraw from the study, you agree that the data collected so far will be analysed anonymously.

In case of need, here are the contact details of the person to contact (Dr Kevin Selby, research manager: telephone number (+41795566753) or e-mail address (kevin.selby@unisante.ch)

YOUR CONTACT DETAILS

Please provide us with your contact details so that the research team can contact you for the questionnaires:

Date : ____

Name : ____

First name : ____

Telephone number : ____

E-mail address: ____

Name of your treating physician : ____

Consent statement for participation in the FIRST study

Please read this form carefully. Do not hesitate to ask questions if you do not understand something or if you need clarification. Your written consent is required to participate in the project.

**BASEC number of the research project (after submission to the relevant ethics committee) :**	2020-02898
**Title**<br>**(scientific and usual) :**	FIRST: The effectiveness of the "vIvRE Sans Tabac" training of treating physicians on the rate of cessation of smoking by their patients
**Responsible institution**<br>**(project leader and full address) :**	Unisanté<br>University Centre for General Practice and Public Health<br>Route de Berne 113<br>1010 Lausanne
**Location :**	Lausanne
**Responsible for the project on the site :**<br>Printed name and surname :	Dr Kevin Selby
**Participant :**<br>Printed name and surname: Date of birth :	

- I declare that I have been informed orally and in writing by the attending physician participating in the study of the objectives and course of the research project as well as the possible advantages and disadvantages and the possible risks.
- I am taking part in this project voluntarily and I accept the contents of the information sheet I have been given about the above-mentioned project. I have had sufficient time to make my decision and sign the consent form.
- I have received answers to the questions I asked in relation to participation in this project. I keep the information sheet and receive a copy of my written consent.
- I take note that the UNISANTE institution that initiated the research project is responsible for its realisation, and is liable for any damage I may suffer in connection with the research activities. The conditions and procedure are set by law.

- I agree that the competent specialists of the project management and the competent ethics commission may consult my uncoded data for the purpose of checks and inspections, provided that the confidentiality of these data is strictly guaranteed.
- I may, at any time and without having to justify myself, revoke my consent to participate in the project, without this decision having any adverse repercussions on my further care. The data collected up to the withdrawal will however be analysed within the framework of the project.

Place, date	Signature of the participant

Attestation of the GP participating in the study (or Attestation of the PI/member of the FIRST study team): I hereby certify that I have explained to the participant the nature, the importance and scope of the project. I declare that I fulfil all obligations in relation to this project in accordance with applicable Swiss law. Should I become aware, at any time during the implementation of the project, of elements that could affect the participant's consent to take part in the project, I undertake to inform him/her immediately.

Place, date	Name and surname of the **GP** participating in the study in block letters. Where Name and surname of the FIRST study team member<br><br>Signature of the **GP** participating in the study Signature of the FIRST study team member

unisanté
Centre universitaire de médecine générale et santé publique · Lausanne

Quitting smoking

CONTENTOFANAIDTOTHEARRET

	MEDICANT	USAGE	PRIX PAR BOÎTE	PRIX PER DAY	EFFICACITY	ADDICTIVITY	PRINCIPALE FFECTS AND SECONDARY
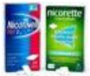	**Chewing gum**<br>Nicotinell / Nicorette ®	**8-12x / day** as needed	~ **20.-** CHF<br>30 x 2 mg gums	~ **5.00** CHF / day	●●	●+	• Irritation of the mouth and throat<br>• Hoquet<br>• Nausea
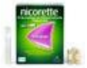	**Inhaler**<br>Nicorette ®	**6-12x / day** as needed	~ **27.-** CHF<br>18 cartridges of 10 mg	~ **9.00** CHF / day	●●	●+	
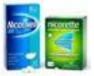	**Tablets**<br>Nicotinell / Nicorette ®	**8-12x / day** as needed	~ **25.-** CHF<br>36 tablets of 2 mg	~ **6.00** CHF / day	●●	●+	
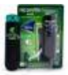	**Mouth spray**<br>Nicorette ®	**12-25x / day** as needed	~ **60.00** CHF<br>150 sprays	~ **6.00** CHF / day	●●	●+	
	**Patch**<br>Nicotinell / Nicorette ®	**1x / day**<br>over 16 or 24 hours or as required	~ **120.00** CHF<br>14 patches of 15 mg	~ **7.00** CHF / day	●●	NO	• Skin irritation
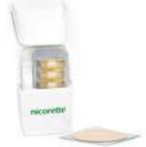	**Combination patch & other nicotine substitute**	**Patch 1x / day**<br>+ other substitute as required	~ **120.00** CHF<br>14 patches and 1 box of substitutes<br>short term	~ **11.00** CHF / day	●●●	●+	• Skin, mouth and throat irritation<br>• Hoquet<br>• Nausea
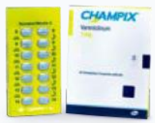	**Varenicline**<br>Champix ®	**2x / day**	~ **120.00** CHF<br>56 tablets of 1 mg<br>● an be covered b y basic insurance under certain conditions	~ **4.-** CHF / day	●●●	NO	• Nausea<br>• Disturbed sleep<br>• Change of mood
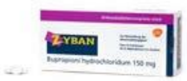	**Bupropion**<br>Zyban ®	**2x / day**	~ **60.00** CHF<br>30 tablets of 150 mg<br>● an be covered b y basic insurance under certain conditions	~ **4.-** CHF / day	●●	NO	• Disturbed sleep<br>• Dry mouth<br>• Headaches<br>• Change of mood<br>• Digestive disorders
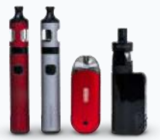	**Electronic Cigarette**<br>Vaporette with nicotine<br>Several brands available	**As required**	~ **50.00** CHF<br>Starter kit – various brands	~ **4.-** CHF / day	●●○	●●●	• Cough<br>• Irritation of the mouth and throat<br>• May expose to toxic compounds in varying concentrations
